# Hybridization of Particulate Methane Monooxygenase by Methanobactin-Modified AuNPs

**DOI:** 10.3390/molecules24224027

**Published:** 2019-11-07

**Authors:** Jia-Ying Xin, Li-Rui Sun, Hui-Ying Lin, Shuai Zhang, Chun-Gu Xia

**Affiliations:** 1 Key Laboratory of Food Science and Engineering, Harbin University of Commerce, Harbin 150076, China; 2 State Key Laboratory of Oxo Synthesis and Selective Oxidation, Lanzhou Institute of Chemical Physics, Chinese Academy of Sciences, Lanzhou 730000, China

**Keywords:** AuNPs, nanobiohybrids, electron donor, hydroquinone, methanobactin, particulate methane monooxygenase

## Abstract

Particulate methane monooxygenase (pMMO) is a characteristic membrane-bound metalloenzyme of methane-oxidizing bacteria that can catalyze the bioconversion of methane to methanol. However, in order to achieve pMMO-based continuous methane-to-methanol bioconversion, the problems of reducing power in vitro regeneration and pMMO stability need to be overcome. Methanobactin (Mb) is a small copper-chelating molecule that functions not only as electron carrier for pMMO catalysis and pMMO protector against oxygen radicals, but also as an agent for copper acquisition and uptake. In order to improve the activity and stability of pMMO, methanobactin–Cu (Mb–Cu)-modified gold nanoparticle (AuNP)–pMMO nanobiohybrids were straightforwardly synthesized via in situ reduction of HAuCl_4_ to AuNPs in a membrane fraction before further association with Mb–Cu. Mb–Cu modification can greatly improve the activity and stability of pMMO in the AuNP–pMMO nanobiohybrids. It is shown that the Mb–Cu-modified AuNP–pMMO nanobiohybrids can persistently catalyze the conversion of methane to methanol with hydroquinone as electron donor. The artificial heterogeneous nanobiohybrids exhibited excellent reusability and reproducibility in three cycles of catalysis, and they provide a model for achieving hydroquinone-driven conversion of methane to methanol.

## 1. Introduction

Green conversion of methane is a growing focus of energy and sustainable development. The only selective catalyst for methane conversion to methanol under normal temperature and pressure conditions is methane monooxygenase (MMO) found in methane-oxidizing bacteria (methanotrophic bacteria) [1]. Methane-oxidizing bacteria can express two different MMOs regulated by copper concentration during growth [2], which are soluble methane monooxygenase (sMMO) and particulate methane monooxygenase (pMMO). In cells cultured under lower copper concentration, the sMMO located in the intercellular space is predominately expressed. In cells cultured under higher copper concentration, the pMMO present in the inner cytoplasmic membrane is exclusively expressed. sMMO consists of three components: a hydroxylase (MMOH, 251 kDa) component composed of three polypeptides and a hydroxo-bridged binuclear iron cluster, a reductase (MMOR, 38.6 kDa) component composed of one polypeptide containing both flavin adenine dinucleotide (FAD) and [Fe_2_S_2_] cofactors, and a regulatory polypeptide (MMOB, 15.9 kDa) [3]. pMMO is a copper-containing enzyme composed of three polypeptides with molecular masses of approximately 45 kDa, 26 kDa, and 23 kDa [4]. The metal content of pMMO reported by different laboratories is controversial, with reported values of 2–15 copper ions and 0–2 iron atoms per ~100 kDa of purified pMMO. With multiple conflicting models proposed for the active site of pMMO, the mechanism of pMMO for methane oxidation is still not clear [2,5]. pMMO is the predominant methane oxidation catalyst in nature and it is particularly attractive for industrial applications because pMMO comprises an estimated 80% of proteins in the inner cytoplasmic membrane [2,5]. Also, the K_m_ for oxygen and methane are quite different, with values of 3 × 10^−6^ mol/L and 1.68 × 10^−5^ mol/L for sMMO, respectively, and approximately 1~2 × 10^−6^ mol/L and 1 × 10^−7^ mol/L for pMMO, respectively [6].

High activity and good stability of the enzyme are prerequisites for the application of biocatalysis. However, as a membrane protein, it is difficult to maintain the activity of pMMO during isolation from membranes [7]. One of the reasons may be the destruction of the electron transport chain of pMMO during the dissociation pMMO from the inner cytoplasmic membrane structure [8]. In the cells of the methanotrophs, pMMO is turned over by reducing equivalents from quinols in the quinol pool within inter cytoplasmic membranes of the organism, as well as from nicotinamide adenine dinucleotide (NADH) or other redox proteins in the cytoplasm. Thus, the electron transport chain is disrupted when pMMO is taken out of the membranes within the cellular environment. Once outside the cell, the methane oxidation activity must depend on other sources of reducing equivalents and the pathway of input of these electrons into the enzyme. Another reason may be the change in the original hydrophobic membrane environment during purification, destroying the native conformation of pMMO. The third reason for the lower activity of pMMO may be the inhibition of reactive oxygen species (ROS). Researchers found that pMMO can aerobically produce ROS in the absence of substrate methane [9]. These results indicate that duroquinol induces ROS formation by pMMO. In the presence of the pMMO substrate, methane, ROS formation was diminished, which is likely caused by the consumption of electrons by methane oxidation [10]. However, the generation of reactive oxygen species (ROS) and its inhibition of pMMO activity could only be found after pMMO was taken out of the cell membrane. In fact, some researchers suggested that pMMO was an oxygen-sensitive protein, and it should be implemented in anaerobic conditions as much as possible [11,12,13]. Also, factors like the weakly solubility of methane and oxygen in water can limit the activity of pMMO isolated from the inner membrane.

An alternative way to obtain activated pMMO catalyst is the acquisition of a pMMO-enriched inner cytoplasmic membrane, rather than removal of pMMO from the inner cytoplasmic membrane. In methane-oxidizing bacteria, pMMO is expressed abundantly in high-surface-area, folded lipid inner cytoplasmic membrane structures that can comprise a large part of the cellular volume [5,13,14]. The inner cytoplasmic membrane provides a more native environment for pMMO. Although weakly soluble in water, methane and oxygen are much more soluble in a lipid membrane [15]. For example, methane is 10 times more soluble in the lipid membrane than in water [15]. Given that methane-oxidizing bacteria evolved to use membrane-bound pMMO as their primary catalyst for methane oxidation, the use of a pMMO-enriched membrane as a catalyst may be a more appropriate and effective strategy.

Unfortunately, a significant decrease in pMMO activity upon isolation of the pMMO-enriched membrane fraction from the cell during cell lysis and ultracentrifugation was reported [14,16]. The exact reason for pMMO’s partial inactivation upon inner membrane isolation is still unknown, but one reason might be the removal of methanobactin–Cu (Mb–Cu) from the native membrane. The activity of pMMO requires the full complement of Mb–Cu. It was reported that Mb–Cu is easily dissociated from the pMMO complex [14,17]. This finding suggests that the loss of pMMO activity upon isolation is due to the removal and loss of Mb–Cu.

Methanobactin (Mb), previously called copper-binding compound, is a small 1154 Da copper-binding peptide with a chemical composition of C_45_N_12_O_14_H_62_S_5_ and a single copper ion coordinated by an N_2_S_2_ donor set, initially identified in the inner cytoplasmic membrane and associated with the pMMO complex [17,18,19,20]. It was reported that Mb can act as an electron storage, transport, and delivery agent, and it is involved in electron flow to the active site of pMMO [21,22,23,24,25]. Mb also serves secondary roles, such as maintenance of a particular redox state and protection against oxygen radicals. It was also suggested that Mb may serve as a regulatory protein [26,27,28]. These unrelated distinct functions of Mb vary with changes in its cell location, physical environment, and complex formation with pMMO, suggesting that Mb may belong to a group of proteins known as moonlighting proteins [29,30]. It was reported that the PmoB subunit is a Cu(I) protein with many copper-binding sites [31]. There is a mononuclear copper site and a dicopper site in the N-terminal sub-domain and “E-clusters” associated with the C-terminal sub-domain of the PmoB subunit [31]. The “E-clusters” provide a reservoir of reducing equivalents to re-reduce the tricopper cluster of the proposed active site after the latter cofactor completes the oxidative phase of the enzyme turnover [31]. The depletion of the “E-clusters” from PmoB would inactivate the enzyme. Mb–Cu was proposed as a part of the E-clusters, which functions as an electron storage, transport, and delivery agent for pMMO and as a reactive oxygen species (ROS) scavenger. Hence, the MMO activity of gold nanoparticle (AuNP)–pMMO nanobiohybrids may be dramatically enhanced if Mb–Cu is modified on AuNPs bound in the inner membrane via an Au–S bond. Most reporting laboratories agree that pMMO is associated with 8–13 Mb peptides and that these Mb peptides should be co-purified with pMMO. The dissociation of these Mb peptides from the pMMO complex would result in the irreversible inactivation of pMMO [13,14].

It was found that Au(III) can be reduced to Au(0), and that Au(0) remains associated with Mb [32,33]. In our previous work, a facile Mb-mediated one-step synthetic route to prepare monodispersed AuNPs was developed [34]. Interestingly, we also demonstrated that Mb can statically adsorb onto the surface of AuNPs via an Au–S bond to form Mb-modified AuNPs [33]. Also, we found that Mb–Cu has peroxidase-like activity with hydroquinone as an electron donor [32].

In the present work, we demonstrate for the first time that it is possible to generate AuNPs in membrane fractions by adding HAuCl_4_ to pMMO-enriched membrane fractions. The residual Mb in the membrane fractions with Au(III) reduction and Au(0) association ability could mediate the in situ formation of AuNPs in the pMMO intramolecular and intermolecular cavities of the pMMO-enriched membranes. In the hybridization, AuNPs were employed as three-dimensional scaffolds for loading the Mb–Cu and pMMO. pMMO and Mb could combine to form a complex due to the inherent affinity, whereby the Mb molecule binds Cu through S and N coordination by the 4-thiocarbonyl-5-hydroxy imidazolate (THI) ring and 4-hydroxy-5-thiocarbonyl imidazolate (HTI) ring. Mb and AuNPs could be joined by an Au–S bond, and pMMO and AuNPs could combine via physical interactions due to the huge specific surface area of AuNPs. A large amount of extra Mb–Cu being reconstituted into the pMMO-enriched inner membrane relied on the large specific surface area and the inherent adsorption of AuNPs onto biomolecules. This reconstitution recovered and increased the pMMO activity. Complex intracellular processes such as electron transfer, reactive oxygen scavenging, and elimination could be easily realized. The Mb–Cu-modified AuNP–pMMO nanobiohybrids could catalyze the direct hydroquinone-driven conversion of methane to methanol.

## 2. Results and Discussions

### 2.1. Preparation and Characterization of AuNP–pMMO Nanobiohybrids

**Figure 1:**
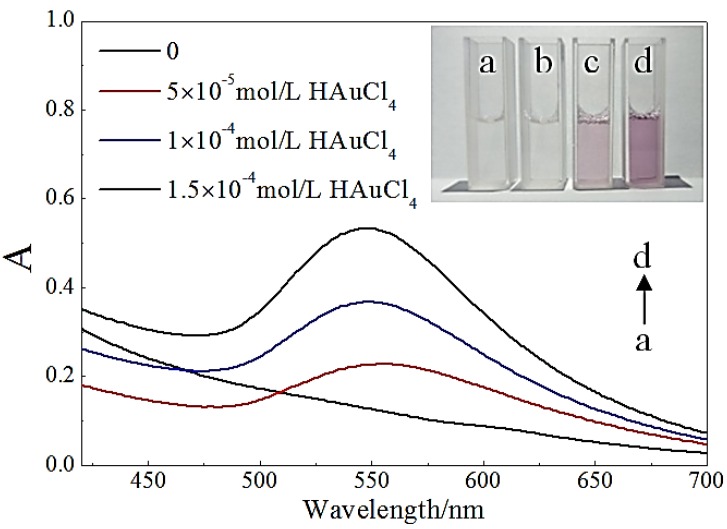
UV–Vis spectroscopy of gold nanoparticles (AuNPs) generated in situ by particulate methane monooxygenase (pMMO)-enriched membrane fraction mediation. The inset shows the different colors with respect to HAuCl_4_ amount.

The preparation of the AuNP–pMMO nanobiohybrids in aqueous medium was carried out by adding the pMMO-enriched membrane fractions to an aqueous solution of HAuCl_4_ and gentle stirring at room temperature. After 24 h, the AuNPs were generated in situ from an aqueous solution of HAuCl_4_. The initial clear solution became a cloudy suspension, forming heterogeneous nanobiohybrids. The nanobiohybrids were recovered by centrifugation at 10,000× *g* for 30 min, washed with distilled water, and lyophilized. X-ray photoelectron spectroscopy (XPS) analysis revealed that 0.45% of Au(III) was entrapped in the pMMO-enriched membrane fractions. As shown in Figure 1, the formation of AuNPs was monitored using ultraviolet-visible spectrophotometer (UV–Vis), and a major peak at 547 nm assigned to the AuNPs was found. It was observed that, with the increase in concentration of HAuCl_4_, the position of the maximum absorption peak blue-shifted, the maximum absorption value gradually increased, and the peak shape gradually narrowed. The blue-shift of the UV–Vis peak can be attributed to the size or chemical environment of AuNPs in the AuNP–pMMO nanobiohybrids. Soluble Au(III) in situ reduced by the residual Mb and the reducing component of the inner membrane fractions created small Au(0) metallic clusters which gradually developed into AuNPs. However, some biomolecules of the inner membrane fractions connected to the surface of AuNPs could prevent the nucleus from getting larger. This indicated that, with the increase in concentration of HAuCl_4_, the content of AuNPs synthesized in situ also increased and the size distribution gradually narrowed, which was accompanied by a change from light yellow to dark red in color.

**Figure 2:**
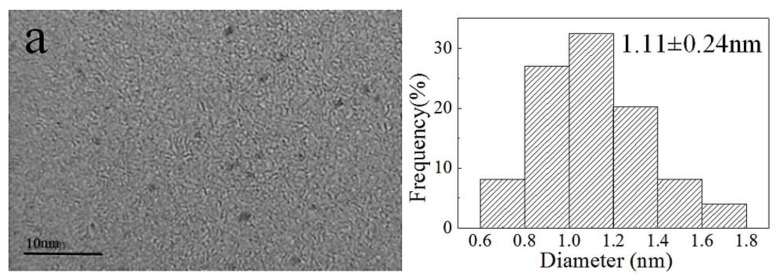

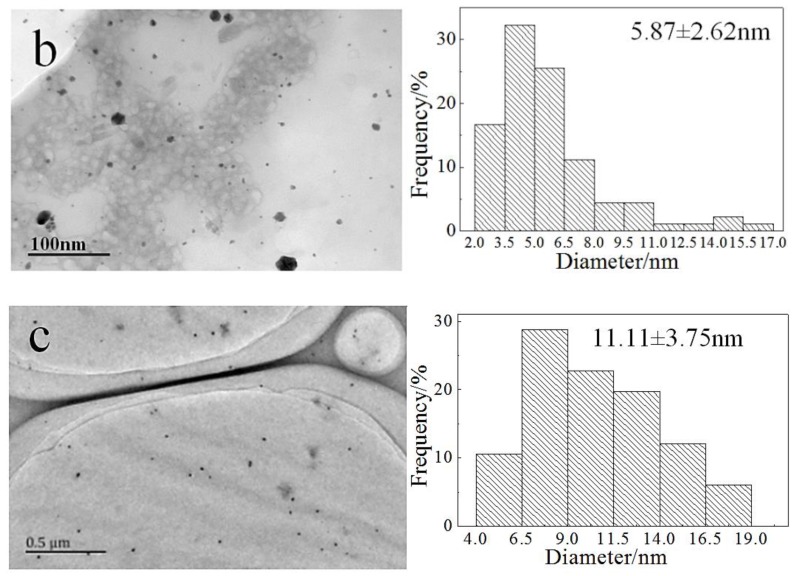
Characterization and size distribution of AuNPs dispersed in the inner membrane organic matrix framework. The concentrations of HAuCl_4_ in a, b, and c are 5 × 10^−5^, 1 × 10^−4^, and 1.5 × 10^−4^ mol/L, respectively.

In these conditions, the AuNP–pMMO nanobiohybrids with an amorphous supra structure composed of AuNPs dispersed and embedded into the inner membrane organic matrix were formed. The generation of AuNPs was further confirmed by TEM and XPS. Figure 2 shows the morphology and the distribution of AuNPs generated in the inner membrane organic matrix at different concentrations of HAuCl_4_. As the amount of HAuCl_4_ added increased, the particle size of AuNPs also increased. When the amount of HAuCl_4_ added was reduced, the main fraction was composed of spherical AuNPs with an average diameter of 1.11 ± 0.24 nm, densely deposited throughout the hybrid composite. When the amount of HAuCl_4_ added was increased, larger AuNPs with an average diameter of 11.11 ± 3.75 nm were randomly decorated in the inner membrane organic matrix. When these AuNPs were enlarged, the crystal lattice of the surface of the AuNPs could be detectable, and the lattice spacing was 0.243 nm. TEM analysis revealed that AuNPs were densely and randomly dispersed throughout the inner membrane organic matrix framework. This implicated that AuNPs might be generated inside or on the surface of the inner membrane.

**Figure 3:**
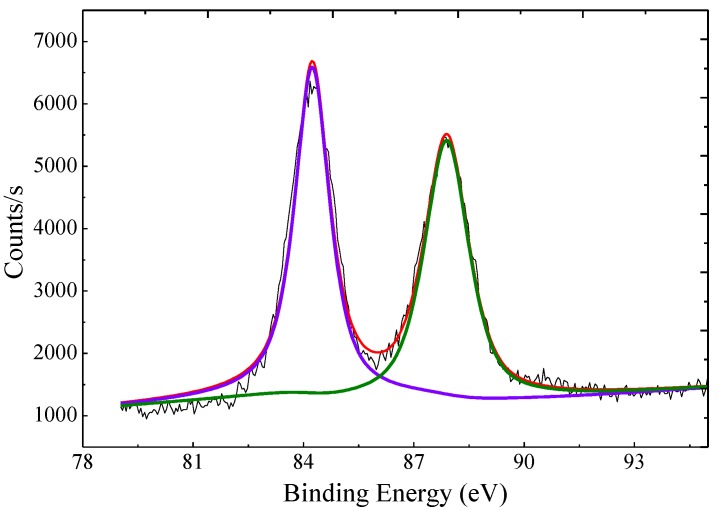
XPS spectra of Au 4*f* regions recorded for the nanobiohybrids.

As shown in Figure 3, the XPS spectrum of the AuNP–pMMO nanobiohybrids showed an Au 4*f*^7/2^ signal around 84.25 eV and an Au 4*f*^5/2^ signal around 87.90 eV, which indicated that Au(III) ions were reduced to the metallic phase. Inductively coupled plasma mass spectrometry (ICP-MS) analysis revealed that Au was entrapped inside the AuNP–pMMO nanobiohybrids with loading amounts from 171.6 μg/g to 473.3 μg/g.

**Figure 4:**
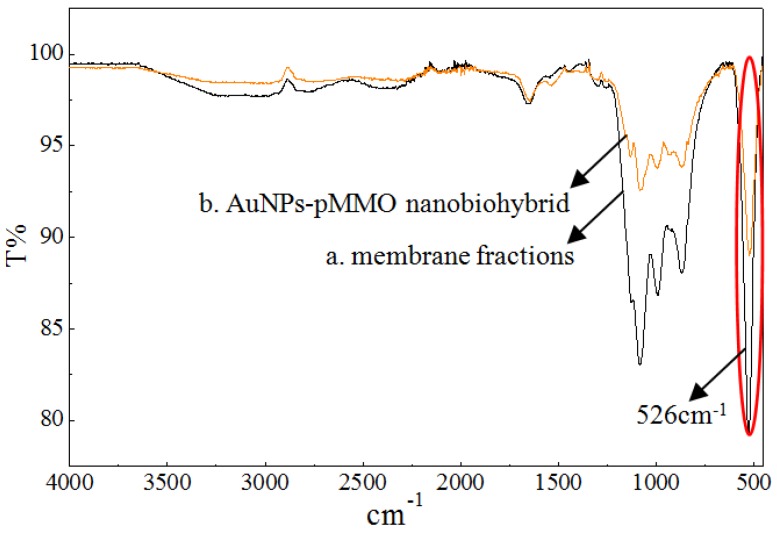
FTIR of membrane fractions (**a**) and AuNP–pMMO nanobiohybrids (**b**).

According to the reported result, the pMMO-enriched membranes with a basic level of pMMO activity contained 8–13 Mb peptides per pMMO complex. The preparation of the pMMO-enriched membrane fraction might have resulted in the partial dissociation of Mb–Cu from the pMMO complex [34]. However, the residual Mb still had Au(III) reduction and AuNP generation ability. Through Mb-mediated reduction of Au(III) to Au(0), the in situ formation of AuNPs would occur in the pMMO intermolecular cavities of the pMMO-enriched membranes. Fourier transform infrared spectroscopy (FTIR) spectroscopy was conducted to compare the difference between the membrane and AuNP–pMMO nanobiohybrids, allowing the identification of functional groups in the inner membrane organic matrix bound to the AuNP surface (Figure 4).

It was found that the weak absorption peak of the disulfide bond at 526 cm^−1^ was obviously weakened in the membrane fractions after the in situ synthesis of AuNPs, which proved that the S–S bond was broken, subsequently forming the Au–S bond. The sulfur species of AuNP–pMMO nanobiohybrids might be different from that of the membrane due to strong interaction between the sulfur and Au species. It was reported that Au(III) can be reduced to Au(0), and then Au(0) can associate to the Mb via an Au–S bond [35]. In this paper, FTIR spectroscopy revealed that AuNPs were probably modified by Mb molecules that had S–S groups, and the association of Mb to the AuNP surface occurred due to the potential interaction between the S–S residues of cystine on Mb and the gold colloid surface. However, the possible Au–S interactions need further confirmation by Raman spectroscopy.

These results confirmed that AuNPs were generated inside or on the surface of the cytoplasmic inner membrane through the action of a reducing amino acid of membrane proteins (or peptides) combined with inner membrane fractions. pMMO and Mb located in the inner membrane were also involved as reactants in the generation of AuNPs and hybrid nanostructures. A proposed plausible nanobiohybrid formation process is postulated below. Soluble Au(III) ions were adsorbed rapidly by the inner membrane fractions through electrostatic interaction and then in situ reduced by the reducing component of inner membrane fractions to Au(0), before becoming a crystal nucleus and gradually developing into AuNPs. AuNPs acted as a cross-linker between pMMO and Mb molecules. At the same time, some biomolecules connected to the surface of AuNPs could prevent the nucleus from getting larger and acted as a protective agent and stabilizer.

pMMO and Mb perhaps involved the formation of AuNP–pMMO nanobiohybirds considering some properties of amino acids and small peptides, as described in the literature [36]. The metal-binding and reducing ability of free amino acids and some small peptides was deeply investigated [36]. It was reported that an ideal peptide sequence must contain amino acids presenting hydrophobic or charged side chains together with neighboring amino acids showing a strong reducing ability [36]. The AuNPs were very stable in aqueous solution without any changes in particle size and morphology for one month (data not shown). This provided further evidence that the membrane network acted not only as a physical support and reducing agent during the AuNP generation but also as a stabilizing agent.

### 2.2. MMO Activity of AuNP–pMMO Nanobiohybrids

**Figure 5:**
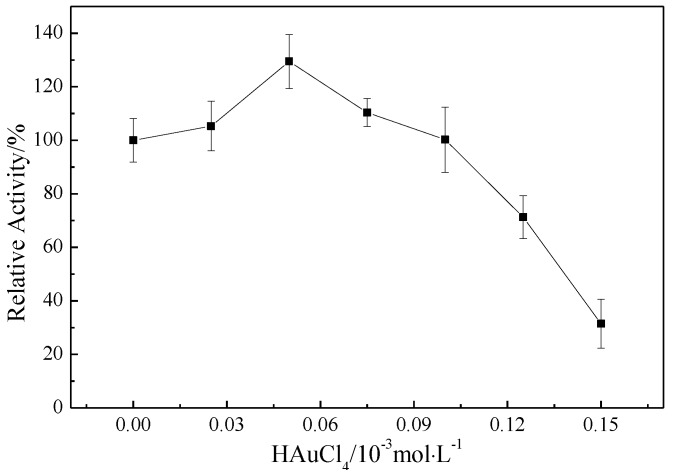
Effect of the amount of HAuCl_4_ on the relative activity of membrane fractions (100% epoxidation activity = 3.57 nmol∙min^−1^∙mg^−1^).

The MMO activity of AuNP–pMMO nanobiohybrids was detected by propene epoxidation. As shown in Figure 5, the MMO activity was found to be strongly dependent on the addition of HAuCl_4_. Upon progressively increasing the concentration of HAuCl_4_ from 0 to 5 × 10^−5^ mol/L, the MMO activity increased gradually. The MMO activity of AuNP–pMMO nanobiohybirds was 26% higher than that of pMMO-enriched inner membrane fractions when the final concentration of HAuCl_4_ was 5 × 10^−5^ mol/L. The possible explanations for this behavior include the peroxidase-like catalytic activity, high electron turnover rate, and high specific surface area of AuNPs. AuNPs might function as a high-efficiency electron shuttle from an electron carrier such as Mb–Cu bound on AuNPs to the metal active center of pMMO, thereby enhancing the reaction rate. However, with a higher concentration of HAuCl_4_, the MMO activity gradually decreased. When the final concentration of HAuCl_4_ exceeded 1 × 10^−4^ mol/L, the MMO activity of AuNP–pMMO nanobiohybrids decreased rapidly. The sharp decrease in MMO activity with HAuCl_4_ concentration increase could be attributed to the interference or disruption of the pMMO natural conformation and metal activity center due to the formation of a large number of AuNPs and a subsequent pH decrease due to HAuCl_4_.

Jv et al. reported that AuNPs with a particle size of 13 nm had peroxidase-like catalytic activity, and smaller AuNPs with greater surface-to-volume ratio had higher peroxidase-like activity [37]. As shown in Figure 2, pMMO in the inner membrane fractions could reduce Au(III) to Au(0) and form AuNPs with an average diameter from 1.11 ± 0.24 nm to 11.11 ± 3.75 nm according to the amount of HAuCl_4_ added. These AuNPs could possess different peroxidase-like activities for scavenging active oxygen molecules such as hydrogen peroxide generated by pMMO. The AuNPs with peroxidase-like activity have certain requirements with respect to particle size. Generally, as the particle size decreases, the peroxidase-like activity of the AuNPs increases. The reduction of hydrogen peroxide reduced the inhibitory effect on the pMMO activity in the inner membrane fractions, leading to an increase in inner membrane fraction activity. Therefore, we studied the peroxidase-like activity of AuNPs in pMMO–AuNP nanobiohybrids.

**Figure 6:**
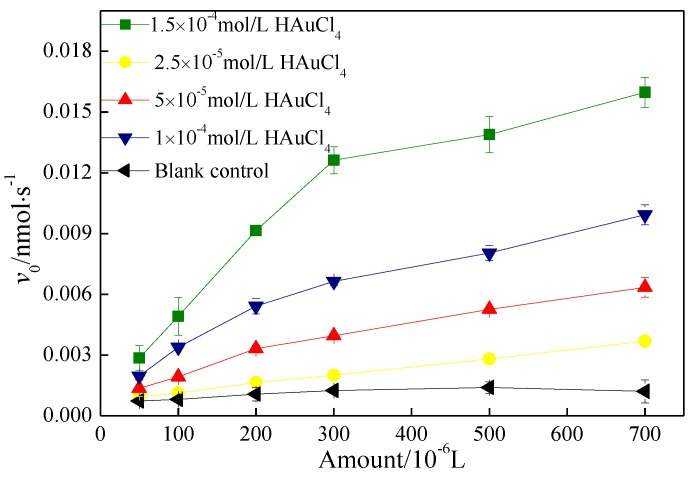
Effect of the amount of pMMO–AuNP nanobiohybrids on the rate of hydroquinone oxidation.

Figure 6 shows the effect of the amount of AuNP–pMMO nanobiohybrid on the rate of hydroquinone oxidation. It was observed that, as the concentration of HAuCl_4_ increased, so did the quantity of AuNPs generated and the rate of hydroquinone oxidization. In addition, the rate of elimination of hydrogen peroxide by AuNPs gradually increased beyond the rate of generation by the inner membrane fractions, leading to an increase in inner membrane fractions activity. However, when the concentration of HAuCl_4_ was too large (>5 × 10^−5^ mol/L), a large amount of pMMO was involved in the synthesis of AuNPs, and the formation of a large amount of AuNPs destroyed the pMMO natural conformation and metal activity center, resulting in a rapid decline in pMMO activity, although the AuNPs in the hybrid enzyme possessed better peroxidase-like activity.

### 2.3. Effect of Mb–Cu-Modified AuNP–pMMO Nanobiohybrids on the Catalytic Activity of pMMO

The structure of Mb following exposure to high copper concentrations featured a molecule folded with a 4-thiocarbonyl-5-hydroxy imidazolate (THI) ring and 4-hydroxy-5-thiocarbonyl imidazolate (HTI) ring in proximity to each other, as well as Cu bound in S and N coordination by two ring moieties. It was noted that Mb–Cu showed reductant-dependent oxidase activity [7]. In our previous work, we also found that Mb–Cu could be used as mimetic peroxidase in the catalytic oxidation of hydroquinone by hydrogen peroxide, and a more significant peroxidase-like activity was exhibited by Mb–Cu-modified AuNPs. As for the Mb–Cu-modified AuNPs, the result suggested that Mb molecules were anchored on the AuNP surface through the disulfide group and could load the Cu catalytic center via free HTI and THI groups [38]. AuNPs might function as an electron shuttle from the electron source to Mb–Cu bound on the AuNPs, thereby enhancing the catalytic reaction rate. Hence, the MMO activity of the AuNP–pMMO nanobiohybrids may be dramatically enhanced if Mb–Cu modifies the AuNPs bound in the inner membrane. To test this hypothesis, Mb obtained from the spent medium was coordinated with Cu and then added to AuNP–pMMO nanobiohybrids to improve MMO activity and stability.

**Figure 7:**
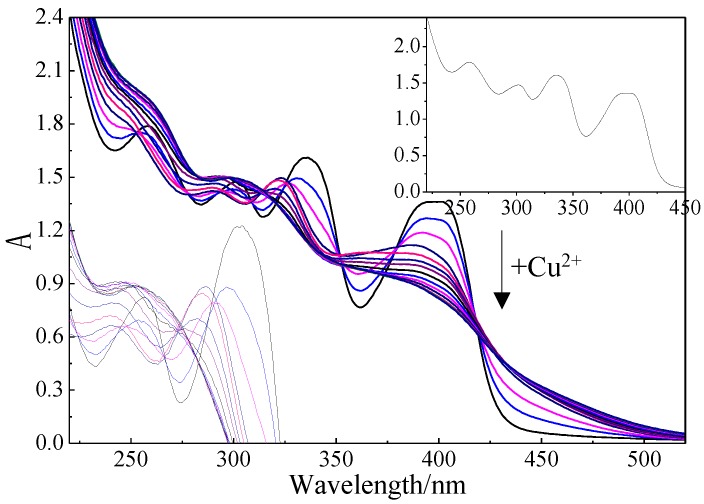
UV–Vis spectroscopy of methanobactin (Mb) following the addition of Cu(II) (0–480 × 10^−9^ mol). The inset panel to the top right shows pure Mb, and the inset picture to the left is a magnified view of the variation of characteristic peaks at 301 nm and 336 nm.

The formation of Mb–Cu by Cu(II) addition was examined by UV–Vis spectroscopy (Figure 7). The UV–Vis spectrum of Mb showed four major peaks at 398 nm, 336 nm, 301 nm, and 258 nm assigned to the 4-thiocarbonyl-5-hydroxy imidazolate (THI) ring, 4-hydroxy-5-thiocarbonyl imidazolate (HTI) ring, tyrosine (Tyr), and cysteine (Cys), respectively. The study found that all four characteristic peaks changed during the preparation of Mb–Cu when Cu(II) was added to the Mb solution. When the main peak no longer changed, it was considered that the coordination of Mb and copper was saturated [31].

**Figure 8:**
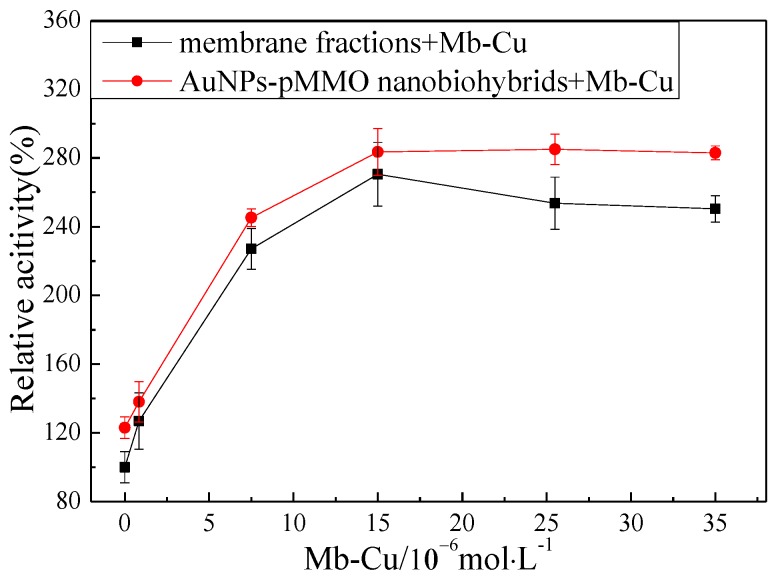
Effect of Mb–Cu concentration on the activity of membrane fractions and AuNP–pMMO nanobiohybrids (5 × 10^−3^ mol/L dimethyl hydroquinone was added to the reaction; 100% epoxidation activity = 3.24 nmol∙min^−1^∙mg^−1^).

In order to investigate the effect of Mb–Cu on the pMMO activity of inner membrane fractions and AuNP–pMMO nanobiohybrids, different concentrations of Mb–Cu were added to the membrane fractions and AuNP–pMMO nanobiohybrids, as shown in Figure 8. The addition of Mb–Cu to the inner membrane fractions had a dramatic influence on the activity of pMMO. Even a small quantity of Mb–Cu could stimulate the pMMO activity significantly. The highest pMMO activity was observed when the concentration of Mb–Cu was 1.5 × 10^−5^ mol/L, with the activity reaching as high as about 240% compared with the blank control. This stimulation was only observed with Mb–Cu. Mb could slightly stimulate pMMO activity. However, the addition of Cu(II) only slightly stimulated pMMO activity at low concentration and obviously inhibited pMMO activity at high concentration.

**Figure 9:**
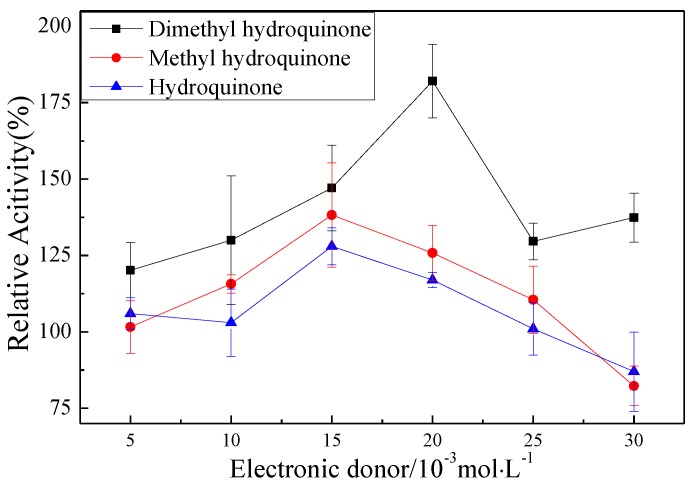
The effect of three kinds of hydroquinone on the MMO activity of membrane fractions (100% epoxidation activity = 2.68, 2.71, and 3.12 nmol∙min^−1^∙mg^−1^).

The promotion of pMMO activity in the membrane fractions by Mb–Cu can be attributed to the electron transport capacity and oxygen species elimination capacity of Mb–Cu. It was found that the addition of hydroquinone to pMMO-enriched membrane fractions resulted in a 24.9% increase in MMO activity, and the activity was increased by 179.8% following the addition of dimethyl hydroquinone (Figure 9). However, Mb–Cu could dramatically improve the activity of pMMO when the reaction was driven by dimethyl hydroquinone. Furthermore, it was reported that Mb–Cu could increase the flow of electrons to the active center of pMMO, suggesting that the role of Mb was to provide a shuttle to transport electrons from the donor to the catalytic center of pMMO [7]. Also, it was reported that Mb–Cu had peroxidase (POD)-like activity and could decrease the number of residual free radicals produced by pMMO.

In the pMMO of methanotrophs, the reducing equivalents come from quinols in the membrane or NADH from the cytosol. Mb–Cu is involved the electron transfer, mediating the electron flow from quinols and NADH in the respiratory chain to the metal centers of the pMMO. Also, by using Mb–Cu as mediator, the exogenous hydroquinone can be used as the reducing equivalent to turn over pMMO [39]. However, the partial dissociation and loss of Mb during the isolation of pMMO-enriched membrane fractions from the methanotrophic cell caused the cleavage of the electron transport chain and weakened the flow of electrons to the active center of pMMO. Therefore, the addition of Mb–Cu could facilitate the delivery of electrons to the pMMO and improve the activity of pMMO. The observed saturation curve of the pMMO activity with respect to the Mb–Cu concentration suggested a specific interaction between pMMO and Mb. These observations provided strong support that the MMO activity of the pMMO-enriched membranes was indeed limited by the dissociation and loss of Mb.

Surprisingly, when Mb–Cu was added to the AuNP–pMMO nanobiohybrids, the activity of pMMO was further improved. Compared with the pMMO-enriched membrane fractions, the activity of AuNP–pMMO nanobiohybrids increased by about 30% (Figure 8).

In our previous study, it was found that AuNPs could obviously enhance the peroxidase-like activity of Mb–Cu. The study suggested that AuNPs supporting multi Cu catalytic centers exhibited an obvious rate enhancement for the catalysis of the oxidation of hydroquinone by hydrogen peroxide. Based on this study, the peroxidase activity of Mb–Cu-functionalized AuNPs was one order of magnitude higher than that of Mb–Cu, which could decompose reactive oxygen species such as hydrogen peroxide produced by pMMO. The Mb–Cu added to the nanobiohybrids led to the development of Mb–Cu-functionalized AuNPs via an Au–S bond between the sulfydryl or disulfide group on the cysteine of Mb and the AuNPs.

**Figure 10:**
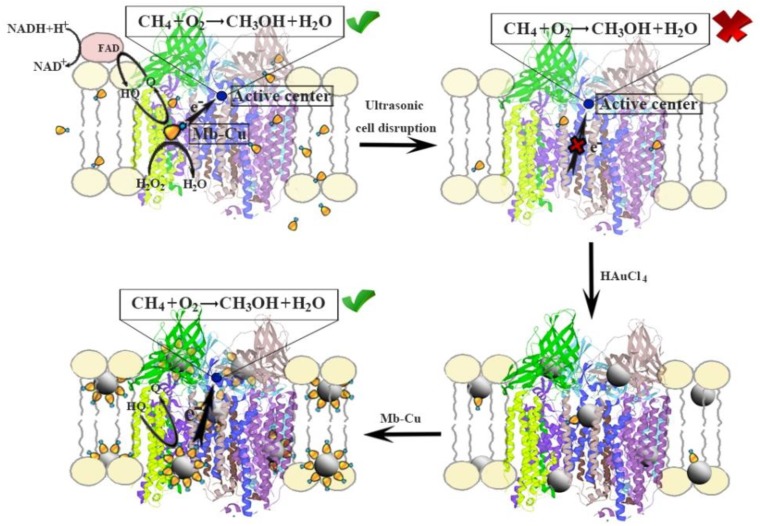
A schematic of the method used to fabricate the AuNP–pMMO nanobiohybrids. The active site is the dicopper site proposed by Rosenzweig and coworkers [11] or the tricopper site proposed by Chan and colleagues [13]).

To further demonstrate that Mb molecules were capped on the surface of the AuNPs, Inductively coupled plasma-atomic emission spectrometry (ICP-AES) analysis was performed to analyze the content of copper. Mb–Cu was added to the hybridase, and after standing overnight, it was centrifuged at 10,000 r/min for 30 min, and the supernatant was removed to obtain Mb–Cu-modified nanobiohybrids. After lyophilization, the content of copper element was measured by ICP-AES and compared with the unmodified nanobiohybrids dissolved in double-distilled water after lyophilization. The results showed that the content of copper in the Mb–Cu-modified pMMO–AuNP nanobiohybrids (571.0 μg/g) was much higher than that of the unmodified nanohybrids, which proved that Mb–Cu was combined with AuNPs in the pMMO–AuNP nanobiohybrids (Figure 10).

### 2.4. Reuse of Mb–Cu-Modified AuNP–pMMO Nanobiohybrids

The pMMO-enriched membrane fractions, the pMMO-enriched membrane fractions with Mb–Cu addition, and the Mb–Cu-modified AuNP–pMMO nanobiohybrids were reused for pMMO activity and stability studies. The pMMO-enriched membrane fractions with or without Mb–Cu addition and the Mb–Cu-modified AuNP–pMMO nanobiohybrids were prepared with the same amount of membrane and then subjected to three cycles of 15-min exposure to methane and oxygen (CH_4_:O_2_ = 1:1, V/V). The membrane fractions or nanobiohybrids were washed thoroughly between cycles to ensure that no residual methanol produced remained. The nanobiohybrids allowed facile reuse following normal-speed centrifugation (10000 r/min), but the membrane fractions were collected by ultracentrifugation (100,000 r/min).

**Figure 11:**
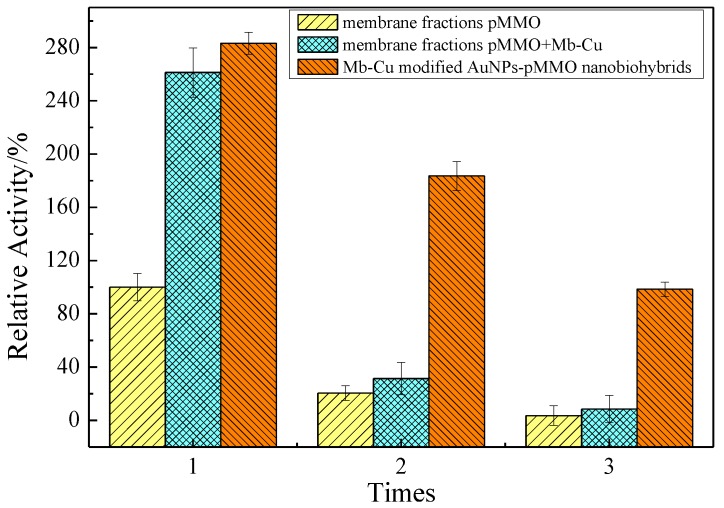
Effect of Mb–Cu on the stability of pMMO in the pMMO-enriched membrane fractions and pMMO–AuNP nanobiohybrids (5 × 10^−3^ mol/L dimethyl hydroquinone was added to the reaction’ 100% epoxidation activity = 2.96 nmol∙min^−1^∙mg^−1^).

The batch reaction stability results of the pMMO-enriched membrane fractions with or without Mb–Cu addition and the Mb–Cu-modified pMMO–AuNP nanobiohybrids prepared with the same amount of membrane are shown in Figure 11. The data indicated that Mb–Cu is an important factor which influences pMMO activity. It was found that the pMMO-enriched membrane fractions were unstable and almost lost all MMO activity after three repeated uses. Mb–Cu addition greatly improved the MMO activity of pMMO-enriched membrane fractions to 260%. However, the stability was poor, and the MMO activity decreased by about 85% after the second repeated use. The reason may be that Mb–Cu and pMMO were combined through to an affinity interaction, which is relatively unstable and, thus, easily decomposed when the external environment changes.

As for the Mb–Cu-modified AuNP–pMMO nanobiohybrids, the small-sized AuNPs synthesized in situ might act as a metal skeleton. On the one hand, AuNPs could tightly bind to the membrane component, and, on the other hand, Mb could modify the AuNP surface via Au–S bond. The Mb–Cu-modified AuNP–pMMO nanobiohybrids performed better in terms of activity and stability. As shown in Figure 11, the activity increased about three-fold compared with the pMMO-enriched membrane fractions with the same amount of membrane. The pMMO activity after the third batch cycle remained close to 34.8% of the initial activity (2.91 nmol∙min^−1^∙mg^−1^). Although more than 65% initial activity of pMMO was lost, the promising results suggested that the Mb–Cu-modified AuNP–pMMO nanobiohybrids could use dimethyl hydroquinone as an electron donor instead of a physiological reductant, and they exhibited improved catalytic activity and stability. This provides a new strategy for addressing the drawbacks of pMMO, such as its easy inactivation, the breaking of the electron transport chain, and its poor stability. Also, the difficulties associated with measuring the methane oxidation activity of pMMO after the protein is taken out of the cell membrane and when the quinols in the quinol pool of the membrane are no longer available could be overcome.

The catalysis capacity of the pMMO-enriched membrane fractions and the pMMO–AuNP nanobiohybrids was tested in the catalysis process for the conversions of methane to methanol and propene to epoxypropane (Table 1). Compared with the pMMO-enriched membrane fractions, the reaction rates of propene epoxidation and methane oxidation catalyzed by the Mb–Cu-modified pMMO–AuNP nanobiohybrids were 2.68 and 2.43 times higher, respectively. By using dimethyl hydroquinone as the electron donor, methane was transformed by the Mb–Cu-modified AuNP–pMMO nanobiohybrids into methanol in almost quantitative yield in 4 h. The nanobiohybrids were reused for three cycles, maintaining their activity.

## 3. Materials and Methods

### 3.1. Chemicals and Apparatus

HAuCl_4_ was purchased from Shanghai No.1 Chemical Co., Ltd. Hydroquinone, dimethyl hydroquinone, ethyl hydroquinone, and all the other chemicals were analytical grade and were acquired from commercial sources. Cell disruption was carried out on a SCIENTZ-II D Ultrasonic cell disruption apparatus from Ningbo Xinzhi Biotechnology Co., Ltd. UV–vis spectral analysis was carried out on a SHIMADZU UV-2550 spectrophotometer from 200 nm to 800 nm at a resolution of 0.5 nm. Ultra-high-speed centrifugation was done on a CS120FNX ultracentrifuge from Hitachi. Epoxypropane was detected by a GC 7900 gas chromatograph from Tianmei Scientific Instrument Co., Ltd., and the quantitative method used was the external standard method.

### 3.2. Microorganism and Culture Conditions

*Methylosinus trichosporium* 3011 was obtained from the Institute of Catalysis (Russian Academy of Sciences) and cultivated according to Xin et al. [38]. Culture conditions for Mb secretion were carried out as previously described [38]. Culture conditions for pMMO expression were carried out under the same conditions, except for the addition of 2 × 10^−5^ mol/L of CuSO_4_ to the medium.

### 3.3. Isolation and Quantification of Mb and Mb–Cu

Mb from the spent medium of the above cultures was isolated as previously described [33,40]. Mb–Cu was isolated using the same methods after incubation of the spent medium of *M. trichosporium* 3011 with excess CuSO_4_ for 10 min. According to the reported result, Cu(II) addition to Mb showed initial coordination with both sulfur and nitrogen, followed by reduction to Cu(I) in less than two minutes [21]. The supernate was loaded onto a Diaion HP-20 column (2.5 × 20 cm, Mitsubishi Chemical Holdings, Tokyo, Japan). The absorbed Mb or Mb–Cu was washed with H_2_O for two to three column volumes and eluted with a mixture of methanol and H_2_O (2/3, *v*/*v*). The eluent was lyophilized, and the freeze-dried samples were used as the source of Mb or Mb–Cu. The amount of Mb in the freeze-dried sample was quantified by the chrome azurol S colorimetric assay [33].

### 3.4. Preparation of pMMO-Enriched Membrane Fraction

Cells were harvested by centrifugation at 8000× *g* for 20 min. Pelleted cells were resuspended and washed in the buffer containing 0.02 mol/L pH 7.2 phosphate buffer saline (PBS) buffer. All isolation steps were carried out at 0–4 °C unless otherwise mentioned. In total, 1 g of wet cells were suspended in 45 mL of 2 × 10^−2^ mol/L pH 7.2 PBS buffer. The cells were broken by a 200-W ultrasonic wave for 10 min, and the cell lysate was then centrifuged at 12,000× *g* for 45 min to remove unlysed cells and cell debris. The supernatant was then centrifuged at 100,000× *g* for 30 min to precipitate the pMMO-enriched membrane fraction. The precipitate was repeatedly washed for three times with PBS buffer to obtain pMMO-enriched membrane fraction.

### 3.5. Activity Assay of pMMO

The pMMO activity of the samples was determined by the propene epoxidation assay. The activity of the pMMO-enriched membranes was used to infer the pMMO concentration. The assays were performed in 10-mL closed vials at 30 °C in a 220 r/min air shock shaker. The reaction was initiated by the injection of 1 mL of propene into the closed vial containing 1 mL of inner membrane fractions or AuNP–pMMO nanobiohybrids. The amount of produced epoxypropane was measured using a gas chromatograph with a hydrogen flame ionization detector and SE-54 capillary column (30 m × 0.32 mm). The carrier gas flow rate was 1.0 mL/min, with a 60 °C injection temperature and 180 °C detector temperature. All measurements were performed at least three times with the same sample. The activity of pMMO was described as the amount of epoxypropane generated from propene.

### 3.6. Assay of Peroxidase-Like Activity

The peroxidase-like activity was evaluated by the rate of oxidation of hydroquinone by hydrogen peroxide. Firstly, hydrogen peroxide and a hydroquinone solution were sequentially added to the cuvette with final concentrations of 1 × 10^−3^ mol/L and 1 × 10^−4^ mol/L, respectively. Next, the AuNP–pMMO nanobiohybrids were added to the cuvette, and the catalytic reaction was carried out at 30 °C. The change of the characteristic peak of hydroquinone at 288 nm was detected by a UV–Vis spectrophotometer at a wavelength of 200–500 nm. The initial velocity of the reaction (*v*_0_) was calculated as follows:

(1) $$v_{0}=\frac{n_{0}-n_{t}}{t},$$

where *v*_0_ is the initial velocity of the reaction, *n*_0_ is the initial content of hydroquinone, *n_t_* is the content of hydroquinone at reaction time *t*, and *t* is the reaction time at which the conversion of the hydroquinone is less than 5%.

### 3.7. Synthesis of AuNP–pMMO Nanobiohybrids

The AuNP–pMMO nanobiohybrids were synthesized by in situ reduction of HAuCl_4_. The residual Mb in the membrane fractions with Au III) reduction and Au(0) association ability could mediate the in situ formation of AuNPs in the pMMO intramolecular and intermolecular cavities of the pMMO-enriched membranes. The reaction was carried out in a 10-mL vial. Different amounts of 5 × 10^−3^ mol/L HAuCl_4_ were added to 1 mL of 30 mg/mL pMMO-enriched membrane fractions with gentle stirring at 30 °C for 24 h. After the incubation, the unreacted HAuCl_4_ was removed by centrifugation (10,000× *g* for 30 min), and the recovered pellet was washed two times with the PBS solution and lyophilized for further physical characterization (FTIR, TEM, XPS, ICP-MS, and ICP-AES).

### 3.8. Characterization of AuNP–pMMO Nanobiohybrids

FTIR spectroscopy was carried out on a Spectrum Two Spectrometer operating at a resolution of 4 cm^−1^ over 4000–450 cm^−1^; the sample for FTIR spectroscopy was lyophilized and ground with KBr to prepare KBr pellets.

TEM was performed on a JEOL JEM-2100F transmission electron microscope. The suspension was spotted on formvar-coated Ni or Cu grids, and the samples on Ni or Cu grids were then dried under vacuum.

XPS was performed using K-Alpha X-ray photoelectron spectroscopy (Thermo Fisher Scientific, USA). Binding energies were referenced to the C 1*s* line at 284.8 eV from adventitious carbon. The samples for XPS were re-dissolved with deionized water three times to get rid of the unattached Mb–Cu molecules.

ICP-AES was performed on an Agilent ICP-OES 730 Spectrometer operating with a plasma gas flow of 15 L/min, an auxiliary gas flow of 1.5 L/min, and an atomizing gas pressure of 200 kPa. The sample for ICP-AES was dissolved in double-distilled water and lyophilized.

## 4. Conclusions

In conclusion, we proposed a new method which can improve the MMO activity of pMMO in membrane fractions via straightforward in situ synthesis of AuNPs to form AuNP–pMMO nanobiohybrids with further modification by Mb–Cu. We replaced or supplemented quinols in the membrane using an external Mb–Cu/hydroquinone (HQ) system to drive pMMO activity. The Mb–Cu-modified AuNP–pMMO nanobiohybrids could use HQ as an electron donor instead of a physiological reductant, and they exhibited improved catalytic activity and stability. The hybridization of pMMO and AuNPs with further Mb–Cu modification could address the drawbacks of pMMO such as its easy inactivation, the breaking of the electron transport chain, and its poor stability. It provides a new reference for the pMMO-catalyzed methane oxidation to methanol.

Although the pMMO-catalyzed conversion of methane to methanol requires hydroquinone to replace NADH as an electron donor, it is a promising application prospect. For example, the photosystem II core complex situated in the thylakoid membrane of cyanobacteria, algae, and plants can catalyze the light-induced transfer of electrons from water to plastoquinone and the release of plastoquinol into the membrane [40,41,42]. It is possible to regenerate plastoquinone by coupling pMMO-catalyzed methane oxidation with the light-driven reduction of plastoquinone. This will eventually realize the photo-driven bioconversion of methane to methanol.

## Figures and Tables

**Table 1:** Specific activity of propene epoxidation and methane oxidation catalyzed by different particulate methane monooxygenase (pMMO) samples. AuNP—gold nanoparticle; Mb—methanobactin.

Sample	Specific Activity (nmol/min·mg)	
	Propene	Methane
Membrane fractions of pMMO	3.16 ± 0.600	2.65 ± 0.770
pMMO–AuNP nanobiohybrids	4.62 ± 1.033	3.37 ± 0.880
Mb–Cu-modified pMMO–AuNP nanobiohybrids	8.48 ± 0.579	6.44 ± 0.837
